# Mechanistic coupling of enzymatic activities at the replisome

**DOI:** 10.1016/j.jbc.2025.110761

**Published:** 2025-09-24

**Authors:** Malisha U. Welikala, Lauren J. Butterworth, Rashini Y. Beragama Arachchi, Michael A. Trakselis

**Affiliations:** Department of Chemistry and Biochemistry, Baylor University, Waco, Texas, USA

**Keywords:** coupling, decoupling, replisome, helicase, polymerase, primase

## Abstract

DNA replication is a vital process requiring the synergistic coordination of enzymatic activities that include unwinding, priming, and synthesis. The helicase serves as the central hub of the replisome and maintains interactions with the enzymes involved in priming and synthesis, which can alter respective structural conformations to control kinetics. This review compares how evolutionarily diverse model systems from phage, *Escherichia coli*, and eukaryotes mechanistically couple these functions to maintain replication stability amid genomic challenges. Despite vast differences in complexity, all systems exhibit conserved principles of coordination through helicase–primase and helicase–polymerase interactions, facilitated by direct binding, intermediary proteins, or conformational constraints. We explore the structural and functional dynamics of replisome architecture, highlighting how core enzymes and accessory proteins collaborate to stabilize and regulate these complexes. Differences in replisome complexity, from the streamlined T7 to more intricate eukaryotic systems, underscore conserved and adaptive strategies for replication regulation. Consequences of replisome blocks leading to stalled forks or decoupled unwinding and synthesis, giving rise to single-strand gaps, are discussed in the context of conserved regulatory responses. Together, we provide insight into universal and divergent replisomal coupling mechanisms, offering a foundation for understanding replication-associated diseases and informing on novel therapeutic approaches.

The DNA replication process involves the coordination and integration of several different enzymatic activities directed by protein subcomplexes within an intact replisome. At the most basic level, the coordination of DNA unwinding, priming, and synthesis activities is essential such that one activity does not dominate another. In theory, the kinetic rates of all enzymatic activities could be synchronized; however, this would not allow for the dynamic regulation needed when specific activities are impacted by genomic obstacles. Instead, the presence of multivalent protein–protein interactions, the ability to alter the conformational state of protein complexes, and the maintenance and sensing of any ssDNA is utilized to ensure the activities within the replisome remain coupled.

Even in the most minimal biological systems, the coordination of DNA unwinding, priming, and synthesis is fundamental in preventing the significant accumulation of ssDNA, which can be labile and prone to breaks (1, 2, 3, 4, 5, 6, 7). Together, the DNA polymerase and helicase enzymes can be physically coupled (*i.e.,* interacting) or allosterically modulated to ensure equal kinetics of both activities. In fact, contacts between DNA polymerases and helicases typically activate and coregulate their respective activities (8, 9). Whether the polymerase restrains the helicase or the helicase drags the polymerase depends on the specific context and more complex structural dynamics across systems. More advanced replisome systems incorporate accessory proteins or posttranslational modifications that direct conformational changes within the hexameric helicase complex, regulating loading, activation, and unwinding to ensure that unwinding does not proceed prematurely (10, 11, 12, 13, 14). This typically includes controlling the dilation and constriction of the central channel of the helicase (15, 16, 17) as well as altering the ring structure between in-plane closed and subunit cracked out-of-plane open conformations (18, 19, 20). Altogether, the helicase conformation will be impacted by the nucleotide binding state, the encircling and binding of DNA, and the interactions of other proteins that can direct loading and activation.

The helicase is central within a replisome organization. It encounters and responds to DNA duplex conflicts at the leading face and coordinates polymerase activities on the unwound template strands. Should the helicase be able to translocate over, across, or through DNA lesions at the leading face, the trailing polymerases may not be able to synthesize over the respective damage and stall. This can initiate the physical decoupling of the polymerase from the helicase (1). Interestingly and importantly, should decoupling occur, phage and bacterial helicases have the ability to dynamically alter their ring structures, possibly to either a closed ring or a dilated ring less competent for translocation (21, 22), slowing its unwinding speed and awaiting the recoupling of the polymerase after traversing the damage. In more complex organisms, the helicase and polymerase have more intimate contacts that allow the temporary disengagement of the polymerase from the template that can be dragged by the helicase around or through any DNA damage, only to reinitiate synthesis downstream. Although this leaves ssDNA gaps, they can be easily filled using multiple polymerase gap-filling mechanisms (reviewed in (23, 24)). The ability to maintain helicase-polymerase coupling even in the presence of translocating strand lesions ensures that the replisome remains intact and continues to function in a continuous motion to complete its synthesis duties.

With the numerous kinetic, structural, and dynamic mechanisms that maintain replisome coupling, it is apparent that persistent or immutable decoupling poses a severe problem for the cell in maintaining genomic integrity. The experimental ability to induce decoupling in engineered cellular systems also allows us to examine which DNA restart or repair mechanisms are utilized to mitigate ssDNA gaps and under what contexts. The combined efforts of structural biology, biochemical reconstitution, engineered genetic systems, and cellular biology reporters will enable us to fully probe the dynamic nature of replisome coupling, the consequences of decoupling, and develop new therapeutics that target this process in both biological and human systems. In this review, we will discuss the mechanisms utilized by diverse species to couple unwinding, priming, and synthesis to maintain a coupled replisome and the consequences and strategies to overcome genomic obstacles that decouple the replisome.

## Coordination of priming and unwinding

**Figure 1:**
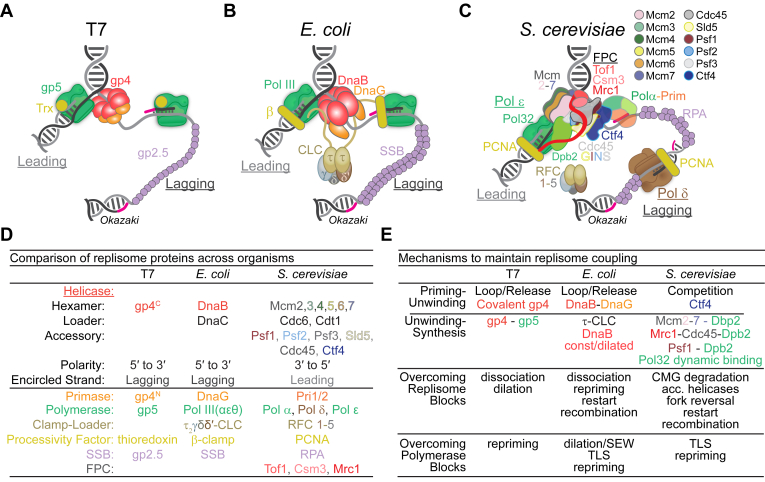
**Replisome architectures from model systems.***A*, the T7 replisome minimally consists of three encoded proteins: gp5, DNA polymerase; gp4, helicase-primase; and gp2.5, single-stranded binding protein as well as the host protein, thioredoxin (trx). *B*, the *E. coli* replisome consists of at least 13 subunits with main complexes: Pol III (αεθ), beta clamp (β), clamp-loader complex (CLC) (δδ′γτ_2_ & ΧΨ), helicase (DnaB), primase (DnaG), and single-stranded binding protein (SSB). *C*, the eukaryotic replisome from *S. cerevisiae* consists of more than 50 proteins within several main complexes: CMG helicase (Cdc45, Mcm2-7, and GINS [Sld5, Psf1, Psf2, Psf3]), leading strand Pol ε (Pol2, Dpb2, Dpb3, Dpb4), lagging-strand Pol δ (Pol3, Pol31, Pol32), PCNA, RFC1-5, replication protein A (RPA), Ctf4 trimer, Polα-Primase (Pol1, Pol12, Pri1, Pri2), and the fork protection complex (FPC) (Tof1, Csm3, Mrc1). *D*, table comparing replisome enzymes and properties across species. *E*, table comparing mechanisms that ensure coupling with specific replisome processes.

Helicases are positioned centrally within the replisome with connections to both the DNA primase and DNA polymerase (Fig. 1). One component primase-helicases system(s) are believed to have evolved from a common evolutionary origin (5, 25) and encode the primase and helicase functions into the same polypeptide as in the Bacteriophage T7 system. Other organisms encode two or more separate polypeptides with separate activities that interact to form a primase–helicase complex, as in bacteriophage T4 or *Escherichia coli*, and may include one or more noncatalytic regulatory subunits, as in the herpes virus or the eukaryotic systems. Two possible scenarios have been proposed for the evolution of these systems (25). The first involves the fusion of the helicase and primase proteins, and the second is the duplication of the ancestral gene containing both helicase and primase properties, where one gene retains the helicase activity and the other gene retains the primase activity. However, the structural homology between the C-terminal domain (CTD) of DnaG primase and N-terminal domain (NTD) of DnaB helicase from *E. coli* suggests that gene duplication from a one component primase-helicase may only apply to the linker region between the NTD and CTD, resulting in one copy of the linker being fused to the N terminus of the helicase and the other copy fused to the C terminus of the primase creating the two component systems (26).

### One-subunit gp4 primase-helicase from bacteriophage T7

While other organisms have separate proteins for the helicase and primase, the T7 bacteriophage primase/helicase, gp4, is unique in that both activities are contained within separate domains of the same polypeptide (Fig. 1*A*). The gp4 gene encodes for two colinear proteins, a 63 kDa protein and a truncated 56 kDa protein formed from an internal start codon with a second ribosome binding site (27). The full-length 63 kDa protein possesses an NTD with associated primase function containing a zinc-binding domain (ZBD) and RNA polymerization domain, a CTD with helicase activity, and a linker region between the two domains required for hexamer formation (28). The 56 kDa protein containing only the CTD helicase domain has a higher likelihood of oligomerization, possibly because the ZBD poses a structural restraint on the oligomerization of the full-length 63 kDa protein (29). Perhaps an excess of ZBDs allows the swapping of the domain structure to cause protomers to be intertwined in a nonfunctional manner to regulate and modulate oligomer stability as also noted for the homologs mitochondrial Twinkle helicase (30, 31). Still, helicase and primase activities performed by the same protein are advantageous, as the primase on its own has a weak affinity for ssDNA binding (10–150 μM). The full-length 63 kDa gp4 has a 100-fold stronger binding affinity owing to the helicase domain interactions with template DNA, effectively localizing DNA priming at sites of unwinding (32).

Expression of the 63 kDa full-length protein is required for T7 propagation, as the 56 kDa truncated form lacking the primase domain is unable to synthesize RNA primers (29). The 56 kDa protein containing helicase activity aids with processive leading-strand synthesis in *in vitro* assays using a steric exclusion strand separation mechanism (33). The 63 kDa protein adds distributive primer synthesis ability with constant association and dissociation from the template (34, 35). However, a mixture of both protein forms (56 and 63 kDa) is more efficient at coupling DNA unwinding and synthesis with the polymerase (gp5) and its processivity factor (thioredoxin [Trx]) than the full-length 63 kDa protein alone (29, 36), suggesting that either a native heterohexamer may contain alternating 56 and 63 kDa subunits with six helicase and three primase domains or alternatively exist as two independent assemblies.

### Two-subunit DnaB helicase and DnaG primase in *E. coli*

DnaB is the replicative helicase in *E. coli*, composed of two domains, linked by a flexible hinge region assembling into a homohexameric complex (37, 38, 39). The CTD of DnaB contains a RecA-like fold with conserved sequence motifs, that is, helix-loop-strand in the core of the fold, characteristic of the SF4 helicase superfamilies to which DnaB belongs (40). The NTD has a fold that is very similar to the C-terminal helicase binding domain of the DNA primase, DnaG (40). DnaG consists of three domains, the NTD which contains the Zn^2+^-binding motif involved in recognizing the specific DNA-binding sequence (41), the central domain which possesses the RNA polymerase (RNAP) catalytic core (42), and the CTD (extreme C terminus, last eight amino acids) (43) which maintains interactions with DnaB used to recruit DnaG to the replication fork (44). The NTD of DnaG sits atop of the CTD of DnaB, but this physical interaction is unstable and fleeting (44), however, it is required for optimal primase activity (45) and DNA priming is stimulated by DnaB (46). Up to three DnaG molecules can bind to a single DnaB hexamer (47) (Fig. 1*B*), analogous to the proposed heterohexamer in T7.

**Figure 2:**
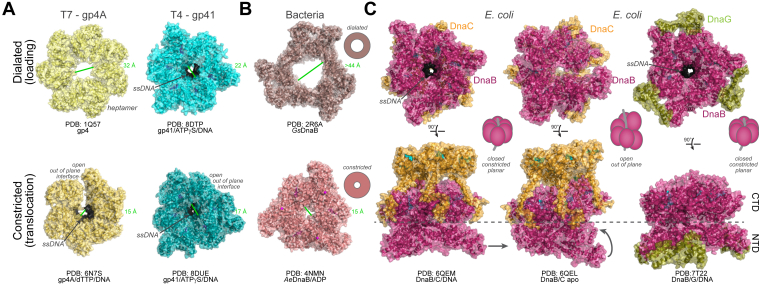
**Constricted–dilated hexameric helicase conformations.** Helicase structures from either (*A*) bacteriophage T7 or T4 or (*B*) representative bacteria showing a dilated states competent for loading (*top*) or constricted planar states loaded and engaging ssDNA (*dark gray*). Pore distances are measured and indicated in *green*. *C*, complexes of either DnaC (closed constricted planar or cracked open out-of-plane) or DnaG (constricted with NTD adjustment) with DnaB at the CTD or NTD, respectively. *Top row* shows the NTD face, while the *bottom row* shows a side view. All PDB IDs are noted along with the compositions of the structures. CTD, C-terminal domain; NTD, N-terminal domain.

Protein interactions with DnaB are not just limited to DnaG, rather, there are other interacting protein partners that can impact the conformation and equilibria of the DnaB–DnaG connections. To load DnaB onto the *oriC* sequence, six DnaC monomers (helicase loader) bind to the RecA fold of the DnaB hexamer in its inactive closed ring state (48, 49). Upon binding of ATP (or other nucleotides), the AAA^+^ domains of DnaC help transition DnaB into the open lock washer conformation, conducive for DNA entry and binding (see Fig. 2 below) (50). This conformational alteration of DnaB by DnaC reduces the binding affinity of the DnaG primase to DnaB at the NTD (51). When DnaC is bound to DnaB, the lengths of the primers synthesized by DnaG are shorter (47). DnaG can initiate primer synthesis while DnaC is still complexed to DnaB; however, full-length primers (11–20 nt) are only synthesized upon dissociation of DnaC from DnaB and unwinding commences (47). Therefore, the proposed model is that DnaG interacting with the NTD of DnaB triggers the dissociation of DnaC from the CTD of DnaB allowing DnaB to transform into a nonplanar state more competent for unwinding and priming (52). This interaction between DnaG and DnaB is an important step in the transition from initiation to the elongation phase of DNA replication (53). For bidirectional replication to begin at *oriC*, RNA primers need to be placed properly (54). Therefore, the interaction between DnaB and DnaG plays a key role in regulating the cycle of Okazaki fragment synthesis (55).

**Figure 3:**
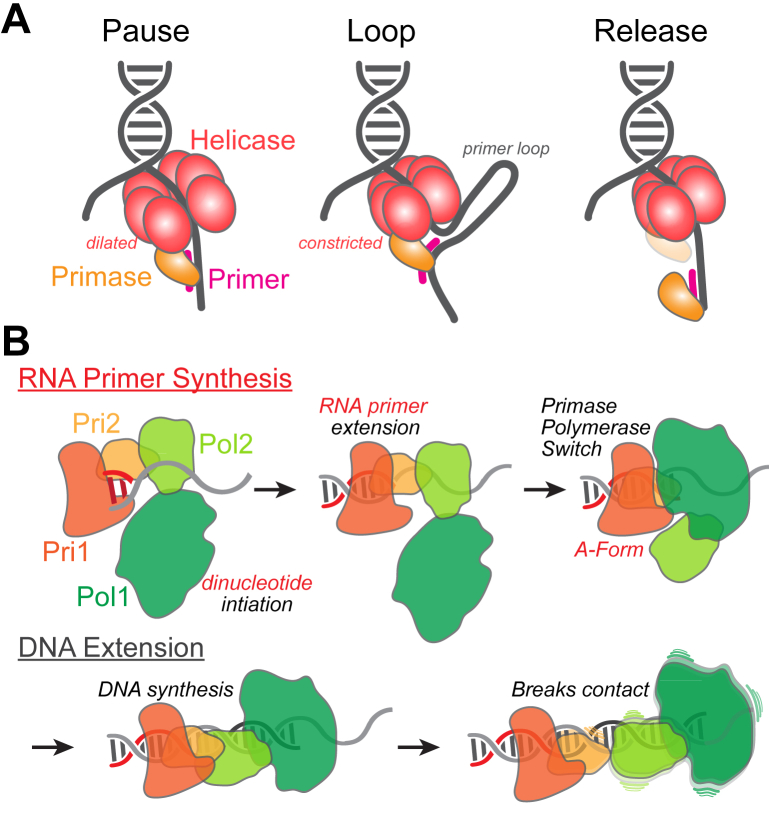
**Mechanisms for coupling DNA unwinding and priming.***A*, physical interactions between the bacterial DNA helicase and primase are required to localize the primase to sites of unwinding. *Pause*, DNA unwinding slows to accommodate DNA priming. *Loop*, a primer loop of various lengths is created to maintain contacts while priming. *Release*, the primase dissociates from the helicase to complete priming independently. *B*, “*competition model*” to control RNA primer and DNA primer lengths for eukaryotic Polα-primase.

The mechanistic coordination between the uninterrupted synthesis of the leading strand and the discontinuous synthesis of the lagging strand has been a topic of intense debate. Previously, it was shown that leading-strand synthesis halts or *pauses* during primer synthesis (Fig. 3*A*) to make up for the delay in the lagging polymerase handover (56). However, later studies in *E. coli* and bacteriophage T4 showed that leading-strand synthesis is not slowed during primer synthesis or during handoff to lagging-strand polymerase. Instead, it was proposed that a priming loop is formed when the primase is physically coupled to the helicase for a quick and efficient handoff of the newly synthesized primer to the lagging-strand polymerase without protein dissociation (57, 58, 59, 60). It was also proposed that the lagging-strand polymerase has a faster DNA synthesis rate than the leading strand polymerase (61), which is governed by the unwinding speed of the helicase and that the DnaG primase can be released from DnaB during synthesis. Therefore, these disparate DNA synthesis rates help the lagging strand DNA primase and polymerase complete ongoing priming and Okazaki fragment synthesis to maintain coupling with leading-strand synthesis (62).

### Eukaryotic Mcm2-7 helicase and the multisubunit Polα-primase

In eukaryotes, priming of freshly unwound ssDNA is carried out by Polα-primase, a four-subunit complex, of which two subunits are associated with catalytic polymerase activity (Pol1/2), whilst the remaining two subunits (Pri1/2) are involved in the DNA priming activity (63, 64). As a result, primer synthesis is a cascade of at least three steps, where the RNA primer is first synthesized, followed by the handover of the RNA primer to the polymerase subunit, and finally, the extension by DNA Polα (Fig. 3*B*). Therefore, unlike in bacteria and phage systems where the RNA primers are short, ranging from 4 to 12 nt, in eukaryotes, the primers are RNA-DNA hybrid molecules with lengths of 20 to 30 nt (65). Priming begins upon transition from G_1_ phase to S, where recruitment of Polα-primase to the replisome is mediated by Mcm10 in initiating chromosomal replication (66, 67). At limiting Polα concentrations, the lengths of the Okazaki fragments synthesized are longer, suggesting that Polα acts in a distributive manner as well as a priming mechanism that might not necessarily be coupled directly with unwinding (68). Similar results of increased Okazaki fragment length were also shown in the *E. coli* system when the concentration of the primase, DnaG, was reduced, consistent with DnaG dissociation and exchange on DnaB (55, 69).

Once recruited to the initiation complex, Pri1/2 will initiate synthesis with an RNA dinucleotide opposite the ssDNA template. Pri1/2 will continue synthesis of the RNA/DNA hybrid up to ∼7 to 10 nt. Remarkably, Pri1/2 will remain bound to the A-form RNA/DNA structure, while a series of complex conformational changes allow Pol1/2 to capture the RNA primer and begin extending a B-form DNA duplex of ∼20 more nucleotides, creating a hybrid primer (70, 71, 72) (Fig. 3*B*) for subsequent extension by the lagging-strand Pol δ. There have been several proposals to reconcile the counting mechanism of Polα-primase. The “*linker model*” suggests that an unstructured region in the middle of Pri2 can act as a ruler between the N and CTDs to spatially constrain RNA primer synthesis (71, 72). The “*steric hinderance model*” was based on structural data that showed a clash between the NTD and CTD of Pri2 that causes Pri1/2 dissociation after synthesis of 9 to 10 ribonucleotides but does not absolutely account for RNA primers seen that are shorter than 8 nt (73). Finally, the “*competition model*” attempts to reconcile the distribution of RNA and DNA lengths synthesized by suggesting that Pri1 and Pol1 compete for the 3′ end of the primer from 6 to 9 nt (70), whereas the “*steric hindrance model”* ensures that primers do not go past 10 nt. Interestingly, the “*competition model*” also revealed that Pri2 remains bound to the A-form RNA/DNA hybrid region after handoff to Pol1/2 and that the connection between Pri1/2 and Pol1/2 becomes successively destabilized as synthesis proceeds up to 20 additional deoxynucleotide bases downstream to limit overall primer length (70).

A physical interaction between Polα and the eukaryotic Cdc45, Mcm2-7, GINS (CMG) helicase is mediated by a replication factor, Ctf4, in budding yeast (Fig. 1*C*) and its human counterpart, AND-1. In yeast, trimeric Ctf4 binds to CMG helicase through the Sld5 subunit of GINS and to Polα through its largest and catalytic polymerase subunit, p180, coupling the activities of both enzymes (74, 75, 76). The C terminus of Ctf4 has six β-propeller domains fused to six α-helices and exists as a trimer with 3-fold symmetry. The Ctf4-binding motif folds into a two-turn α-helix and docks into the protruding helical region of the Ctf4 protomer (76, 77). The N-terminal tail of the Sld5 subunit of GINS contains the Ctf4-binding motif with a similar pattern of conserved hydrophobic residues that are present in the Ctf4-binding motif of Polα. Therefore, Ctf4 can bind to the CMG helicase and two Polα complexes, mimicking a functional resemblance to the *E. coli* replisome, which also has multiple polymerases tethered to the replisome for recycling of leading- and lagging-strand synthesis. Alternatively, the Ctf4 trimer could also localize two CMG complexes and a bridging Polα/primase, resembling a replication factory. Interestingly, the Ctf4/primase interface is positioned on the leading N-terminal face of the CMG helicase in close proximity to the immediately excluded lagging-strand template, analogous to the bacterial replisome where the primase is also engaged with the trailing N-terminal face of DnaB having immediate access to the separated lagging strand emerging from the central channel of the hexamer (Fig. 1, *B* and *C*).

Unlike its homologs, *AND-1* from humans, *mcl1* from *Schizosaccharomyces pombe,* and *sepB* from *Aspergillus nidulans*, *ctf4* is not essential for cellular viability in budding yeast. This may be because in yeast, Mcm10 plays a stronger role in stabilizing Polα, whereas in humans, AND-1 is more directly involved in stabilizing POLα. Therefore, the presence of additional interactions in yeast that help stabilize the polymerase would explain why Ctf4 is not essential for viability (76). Although *in vitro* studies have shown that Ctf4 has a minimal effect on priming on either strand, *in vivo* studies show that Ctf4 stimulates recruitment of Polα for lagging-strand initiation. This can either be from the recruitment of Polα to the fork by Ctf4 and increasing the availability of Polα or because the trimeric structure of Ctf4 can bind to three partners at a given time: two Polα complexes and the helicase, and thereby tethered to the replisome (68, 74).

## Coupling of unwinding and synthesis

Replication systems across all Domains of life have evolved to facilitate dynamic interactions between the helicase and polymerase enzymes to coordinate unwinding and synthesis activities either through direct physical interactions, intermediary partners, or conformational restrictions (Fig. 1). By coupling the activities of both major enzymes within the replisome, unwinding and synthesis can be performed in tandem, without interruption, and regulated in response to any genomic challenges.

### The *gp4 helicase* and gp5 polymerase in the T7 system

The minimalistic nature of the T7 bacteriophage replication system makes it an ideal system for studying essential replisomal coupling interactions. Proteins gp4, gp5, and gp2.5, along with the host processivity factor, Trx, are the only four proteins required for replication (Fig. 1*A*). The polymerase, gp5, belongs to the A-family of polymerases and has both polymerase and exonuclease activity, resembling *E. coli* Pol I (78). gp5, on its own, has low processivity and can only catalyze extension ranging from 1 to 50 nt before dissociating (79); however, a processivity factor, Trx, encoded by the host *E. coli*, increases the processivity of the polymerase for >1000 s of nucleotides (79). Trx also increases the stability of the polymerase–primer–template complex by 20- to 80-fold (80). Trx binds weakly to gp4 and gp2.5 directly but stimulates the binding of gp5 with both gp2.5 and gp4 (81).

Translocation along DNA by the T7 helicase, gp4, occurs through a “hand-over-hand” mechanism, where the helicase tier adopts a cracked open lock-washer conformation encircling the ssDNA and the individual subunits take turns around the ring, translocating up and along the duplex for unwinding. Five of the six domains of hexameric gp4 are in an ordered state, while the sixth domain is flexible and out-of-plane, ready for translocation. gp4 translocates on ssDNA in the 5′ – 3′ direction with each flexible domain stepping 2 nt for each deoxythymidine triphosphate (dTTP) hydrolyzed (82). The gp4 helicase translocation rate along ssDNA is ∼300 nt/s but reduces to ∼22 nt/s on dsDNA. Behind the helicase within the replisome, the leading strand T7 polymerase, gp5, on its own can synthesize DNA with a rate of ∼150 nt/s but with a processivity of only 5 to 6 nt when acting on a dsDNA substrate and performing strand displacement synthesis (6), but it has a much greater processivity (∼800 nt) and increased translocation rate (∼200 nt) when working on a ssDNA substrate (83). When gp4 and gp5 enzymes are kinetically coupled, they can achieve a combined strand displacement and synthesis rate of ∼150 nt/s with significantly increased processivity of 17 kilobases. It is interesting that the polymerase stimulates unwinding of the helicase by pushing it along, but the helicase also helps lower the *K*_m_ of dNTP binding to the polymerase to increase its catalytic rate (84). The combined stability of both enzymes reduces their off-rates from DNA, thereby effectively increasing their combined and coupled kinetic rates.

**Figure 4:**
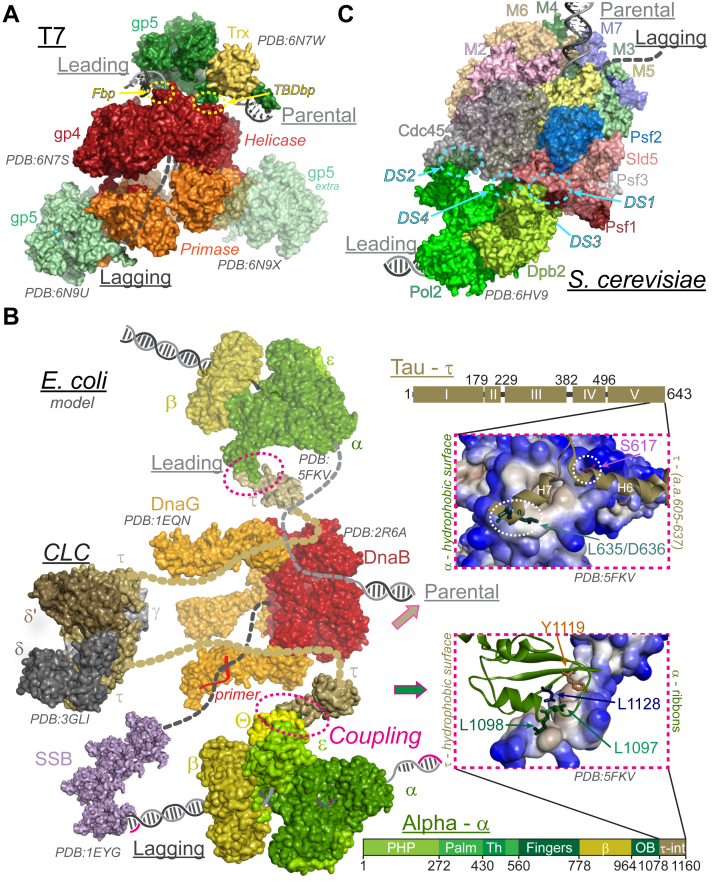
**High resolution helicase-polymerase coupling complexes and models.***A*, replisome model from bacteriophage T7 (PDBs: 6N7S, 6N7W, 6N9U, 6N9X) (82) highlighting physical coupling interactions between the helicase and polymerase. The two basic patches for T7 gp5–gp4 interactions (*Fbp* and *TBDbp*) are indicated with *dashed circles* (*yellow*). *B*, model of the *E. coli* replisome created from X-ray crystal structures of DnaB (PDB: 2R6A, *red*), DnaG (PDB: 1EQN, *orange*), Pol III-β-τ (PDB: 5FKV, α, ε, θ, *green*; β, *cyan*; τ, *taupe*), γ-CLC (PDB: 3GLI, δ, *steel*; δ′, *brown*; γ, *gray*; τ, *taupe*), and SSB (PDB: 1EYG) that physically couples unwinding and synthesis (*pink dashed box*). Unstructured domain IV of τ is shown as *dashed ovals* (*taupe*). Insets show close ups of the proposed coupling interactions of domain V of τ, residues 605 to 637 (*ribbons above, hydrophobic surface below*) interacting with α (*hydrophobic surface above, ribbons below*) is highlighted. Residues indicated in τ (S617, *pink*; L635/D636, *teal*) or α (L1097, *emerald*; L1098, *green*; L1128, *navy*; Y1119, *crema*) were shown to be important for coupling (4, 5). Linear protein schematics of domains for τ (domains I–V) or α (PHP, polymerase and histidinol phosphatase, Palm; Th, thumb; Fingers, β-binding; OB, OB-fold; τ-int, τ interaction region) are shown below each inset or (*C*) replisome structure from *S. cerevisiae* (PDB: 6HV9) (117) showing several docking sites (DSs) that detail the interaction of yeast Pol ε with CMG (*cyan, dashed circles*). The Dpb2 interacts with Psf1 of the GINS complex (DS1) and Mcm5 (DS3, *back side*). The Pol2 CTD interacts at the interface between Mcm2 and Cdc45 (DS3), while the ZF2 motif interacts with Mcm5-CTD (DS4). The catalytic subunit, Pol2, is swung away from optimal positioning for leading strand synthesis in this loading state. CLC, clamp-loader complex; CTD, C-terminal domain; SSB, single-stranded binding protein.

gp4–gp5 coupling is not just restricted to enhancing each other’s kinetic activities; the two enzymes are also physically coupled (Fig. 4*A*). The acidic C-terminal tails on the gp4 helicase domains interact with basic patches on the gp5 polymerase. One basic patch is near the Trx binding domain (TBDbp), and the other is at the front, close to the separation pin (Fbp) (82). The interaction of gp4 with TBDbp is important for polymerase exchange, and the interaction with Fbp is important for loading (85). The polymerase is facing the C-terminal end of the helicase at the fork, which enables electrostatic interactions between the negatively charged C-terminal tails of gp4 and positively charged patches on gp5. These interactions of both enzymes at the fork aid in the functional and kinetic coupling of helicase and polymerase (6).

The parental duplex DNA lies between the helicase and leading strand gp5 polymerase, and the separated lagging strand threads through and down the central pore of gp4 helicase to resemble a T-shape (Fig. 4*A*). The polymerase has a beta-hairpin loop (β-loop) facing the fork opening and a residue, W579, stacked against the first base pair of parental DNA that aids in strand separation. The destabilizing force applied on the fork by the polymerase and the two separated DNA strands being pulled apart in opposite directions enables the helicase to operate at optimal activity (86). In mutant gp4 helicases lacking C-terminal tails (gp4-ΔC17), which interact with gp5, the processivity reduces from 17 kb to ∼5 kb (87). Therefore, this electrostatic coupling interaction is crucial in maintaining the polymerase's tethering to the replisome, even when it dissociates from the leading strand.

In T7, the leading strand polymerases are frequently exchanged with other enzymes in solution in a concentration-dependent manner. In the presence of excess polymerases at the fork, each gp4 monomer can associate with extra gp5 polymerases in solution (Fig. 4*A*, *transparent gp5, extra*) and exchange these polymerases with the one actively synthesizing the leading strand should it dissociate from the 3′OH (88). While these dynamic helicase–polymerase interactions can mediate polymerase exchange to increase processivity, it is also useful when replisomes encounter obstacles and need to bypass them to prevent fork collapse (62).

### The DnaB helicase and Pol III in *E. coli*

DnaB unwinds dsDNA utilizing the power of nucleotide hydrolysis to translocate along the lagging strand in the 5′ to 3′ direction, with the CTD oriented toward the replication fork (Fig. 4*B*) (89). While much of the bacterial replisome is highly dynamic, the helicase serves as a stable platform upon which the other replisomal enzymes freely exchange. In the cell, each DnaB complex can accommodate two tau (τ) promoters from a single clamp-loader complex (τ_2_γ-CLC), where each τ binds one leading and one lagging-strand Pol III core (αεθ) (90). It is also possible that CLC can contain three τ-subunits (τ_3_-CLC), lacking γ, allowing for even more frequent Pol III localization and exchange (91). The τ-subunits within the CLC are proxies through which Pol III and DnaB interact and couple their enzymatic abilities. Coupled DnaB-mediated unwinding and Pol III synthesis achieves maximal rates of 1000 bp/s in the presence of τ, but when the physical interaction between τ and either DnaB or Pol III is eliminated by using γ_3_-CLC or specific targeted decoupling mutations in DnaB, τ, or α-Pol III in *in vitro* assays, there is a ∼20-fold reduction in leading-strand synthesis (2, 4, 5).

τ-Subunits contain five domains, where domains I to III form the core of the CLC, while domains IV and V are required for physical contact and communication to both DnaB and Pol III, respectively (Fig. 4*B*). Specifically, residues 430 to 498 within domain IV of τ bind DnaB (90), but the exact complementary residues on DnaB are currently unknown and are likely located somewhere within the CTD. The Pol III core forms a heterotrimeric complex containing theta (θ), which acts to stabilize the core (92, 93), epsilon (ε), which is the 3′ to 5′ exonuclease proofreading subunit (92), and alpha (α), which contains the polymerization active site. On α, there is a stretch of residues in the extreme C terminus (1078–1160), deemed the τ-interaction region (τ-int), which has been visualized by cryo-EM (94). Residues 611 to at least 636 in domain V of τ interact with the C terminus of α-Pol III through hydrophobic surface interactions (Fig. 4*B*) (95, 96, 97). Recently, several of these interfacial residues on either τ or α have been mutated and shown to disrupt replisome coupling in *E. coli*, causing complex and gross genomic instabilities (4, 5).

Replisomal coupling interactions are frequently disrupted and reestablished during enzyme exchange from various genomic obstacles or as a result of necessary repriming throughout the replication process. Several hexameric (or heptameric) helicase structures (*e.g.,* T4 and T7) have been solved that show various conformations of i) a closed dilated state more consistent with unbound or prebound intermediates, ii) a closed constricted planar state, existing in an inactive ssDNA loaded state, or iii) a more cracked open out-of-plane conformation engaging ssDNA in the central pore and competent for translocation (Fig. 2*A*) (82, 98, 99). Binding of dTTP to the T7 gp4 helicase induces a conformational change in the structure to allow for stronger binding of additional dTTPs involving intersubunit cooperativity that facilitates translocation in the open out-of-plane state (100, 101). X-ray crystal structures of DnaB from thermophilic bacteria showed that DnaB can also exist in at least two inactive closed planar conformations, an extreme dilated conformation with a wide central pore and a constricted conformation with a narrow central pore (Fig. 2*B*). Validation of the dilated state in *E. coli* was confirmed only in the absence of any nucleotide (16). The binding of ATP, various nucleotides, or nucleotide analogs induce the DnaB hexamer toward the constricted out-of-plane form more capable of rapid unwinding. However, even at high nucleotide concentrations or with the addition of ssDNA, DnaB still exists in an equilibrium between closed and cracked-open states, indicating that the alteration of the hexameric structure is likely important for regulating unwinding.

DnaC loads DnaB onto ori regions, proceeding through a cracked open out-of-plane conformation before depositing DnaB/C complex around ssDNA in a constricted closed planar conformation (Fig. 2*C*) (10). DnaC dissociates, allowing DnaG to engage with and further rearrange the NTD of DnaB (72, 73), possibly between a planar closed and a cracked open out-of-plane conformation (60). In either case, there is strong evidence that the conformational state of DnaB induced by DnaG binding also strengthens the interactions with τ-CLC (16, 60). In this DnaG/τ-CLC–induced conformation, the DnaB helicase has a narrow central pore but may alternate between open out-of-plane and closed planar conformations to unwind dsDNA at speeds up to 1000 nt/s in the replisome (102). However, the presence of τ-CLC alone can still allow DnaB to dynamically adjust its inner pore size for either a dilated state, accommodating transversing over duplex DNA, or a constricted state, translocating on ssDNA for enhanced unwinding (4).

When a polymerase becomes stalled (*i.e.*, at a lesion or other genomic block) and must be exchanged, the polymerase loses its connection to the helicase as mediated by τ. This transient decoupling increases the size of its central pore, inducing a closed constricted or dilated state (10) which slows the unwinding rate to <100 nt/s (89), effectively allowing synthesis to catch up to unwinding to reestablish replisomal coupling. Notably in the slower conformational state, the exterior surface of DnaB can also interact with the excluded leading strand in a steric exclusion and wrapping mode (103) to block τ binding until the connection with Pol III has been restored. When site-directed CRISPR/Cas9 genomic editing was used to introduce mutations inducing perpetual constricted states of DnaB with fast unwinding, severe cellular and genomic repercussions resulted including viable and fit strains, increased chromosome complexities, unstable genomes, increased mutational frequencies, and prevalent single-strand gaps (2). It was found that the single-strand gaps were mitigated by SOS and RecA binding that increased the mutagenicity in these strains (3). Therefore, the unwinding speed of the DnaB helicase is multidimensionally regulated i) through a direct connection to τ indirectly connecting the leading strand Pol III core (Fig. 4*B*), ii) through conformational switching between dilated-constricted or open-closed states to control the unwinding rate, iii) through exterior interactions with any excess leading strand ssDNA that stabilize the dilated state, and iv) through contacts with other interacting proteins that impact the conformation state (3, 16, 89, 104, 105).

### The CMG helicase and polymerase connections in eukaryotes

Even though the eukaryotic replication machinery has evolved into a more complex and dynamic system with several more levels of regulation than the prokaryotic systems, interestingly, the helicase–polymerase coupling interaction is more direct and occurs along the same leading strand. The CMG complex serves as the main replicative helicase in eukaryotes, translocating along the leading strand in the 3′ to 5′ direction and proceeding NTD-first with parental and leading strand DNA within the overall replisome resembling a J-shape structure (Fig. 4*C*) compared to the T-shape structure of T7 (Fig. 4*A*) (11, 106, 107). The CMG complex is assembled at the onset of S phase, where the Mcm2-7 helicase is activated through phosphorylation by cyclin-dependent kinases and Dbf4-dependent kinase (108, 109, 110). Cdc45 and GINS are crucial for the full activation of the CMG helicase and closing of the Mcm2-Mcm5 gate (111, 112, 113). ATP hydrolysis by Mcm2-7 is required to complete the loading process and translocate away from the loading factors to form the double hexamer (114, 115). Afterward, the leading strand polymerase epsilon (Polε) binds to form the CMG polymerase epsilon (CMGE) complex that can begin coupled unwinding and synthesis at a rate of ∼50 nt/s, about 20-fold slower than in bacteria, owing to a greater complexity of the replisome complex (Fig. 4*C*) as well as acting in the context of chromatin (116, 117, 118, 119).

#### CMG polymerase epsilon

Polε is the primary replicative polymerase for leading-strand synthesis in eukaryotes (120), and its coupling with the CMG helicase is crucial for efficient and continuous replication (121). Most of the structural characterizations of the Polε and CMG have been done in the yeast system, although emerging research is finding similarities and subtle differences in the vertebrate *Xenopus* and human systems (122, 123, 124). Yeast Polε is a heterotetramer consisting of a Pol2 catalytic subunit and three noncatalytic subunits of Dbp2, Dbp3, and Dbp4. The CTD of Pol2 (POLE1 in humans) subunit also contains a catalytically inactive polymerase module that interacts with Mcm2 and Mcm5 subunits, while the Dbp2 (POLE2 in humans) subunit of Polε interacts with Psf1 of GINS and Mcm5 to effectively keep the Mcm2-5 gate closed at the CTD (Fig. 4*C*) (125, 126).

Further cryo-EM structural studies have elucidated the detailed mechanism of Polε′s interaction with the CMG helicase (117, 127). Polε subunits bind to the CMG complex through four distinct docking sites (DSs) (Fig. 4*C*). The first DS (DS1) involves the NTD of the noncatalytic Dpb2 subunit binding to the B-domain of Psf1 from the GINS complex, a critical interaction for incorporating Polε into the replisome in yeast. The remaining DSs involve more direct interactions with the Mcm motor domains. At DS2, the CTD of Pol2 interacts with the interface between Mcm2 and Cdc45, primarily through hydrophobic contacts. At DS3, the Dpb2-OB domain binds to the CTD interface of Mcm3 and Mcm5. Together, DS2 and DS3 effectively keep the Mcm2-5 gate closed at the CTD. At DS4, the ZF2 domain and the dead polymerase fold of the Pol2-CTD interact with Mcm5-CTD. While the Mcm5-WHD domain participates in interactions at DS4, it is not essential for Polε′s docking onto the MCM ring, demonstrating the dynamic flexibility of the CMG complex. This series of interactions ensures the tight coupling and retention of Polε with the CMG helicase, facilitating synchronized DNA unwinding and leading-strand synthesis, and enhancing the processivity of leading-strand synthesis (118, 128).

The N-terminal catalytic domain of Pol2 is essential for high-speed replication, but it has been shown that Dpb2 alone can support helicase assembly and replication *in vitro*, albeit at a slower rate from suboptimal 10.13039/100015908CMG activation (129). In the absence of Polε′s catalytic activity, Polδ can substitute for leading-strand synthesis, although this substitution significantly compromises replication efficiency (118). It was recently reported that the noncatalytic CTD of human POLE1 (Pol2 in yeast) can partially support DNA replication, suggesting conserved noncatalytic functional interactions with CMG across species (129).

At the front of the CMGE complex, binding the preceding parental DNA duplex, a fork protection complex comprising of CLASPIN (Mrc1 in yeast), Timeless (Tof1 in yeast), and Tipin (Csm3 in yeast), directly interacts with the CMG helicase, spanning both the Mcm2-7 N- and C-terminal tiers (130, 131). These interactions are important to modulate CMG helicase activity and limit backtracking, thus stabilizing fork progression (132). Interestingly, CLASPIN (or yeast Mrc1) also binds to POLε (133, 134) at the opposite end of the CMGE complex through a long intrinsically disordered region that makes several contacts along the length of CMGE. Mrc1 is heavily phosphorylated and modulated in response to DNA replication stress, which controls the efficiency of synthesis by Polε (135, 136, 137). Therefore, CLASPIN (Mrc1) further enables synchronization and coupling of helicase activity with DNA synthesis in eukaryotes (124).

Mechanisms to regulate the speed of DNA unwinding by eukaryotic CMG have not been described to the same degree known for bacterial DnaB. CMG has many more surface interacting proteins that likely restrict any significant conformational changes within the ring structure as seen for phage and bacterial helicases. However, it is known that the Mcm2-Mcm5 gate (138), important for loading and activation at origins (139, 140), can also transiently open during unwinding to transition over and onto parental duplex DNA, effectively halting unwinding when it becomes uncoupled from the polymerase (12). It has also been shown in some cryo-EM structures that a short stretch of parental duplex can enter the NTD of the Mcm2-7 central channel, where the authors suggest that unwinding can take place inside instead of outside in a “*dam-and-diversion*” mechanism (141). However, it has been shown subsequently that the inclusion of replication protein A in these *in vitro* assays may prevent this duplex mode of interaction and instead stimulate CMG unwinding solely by a steric exclusion mechanism (142, 143).

On the other C-terminal end of CMG, the interacting Polε subunits can alternate engagement with the AAA^+^ domains of Mcm2 and Mcm5, likely during the rotational translocation of the helicase along the leading strand (127). Polε maintains its connection to CMG primarily through the Psf1 and Cdc45 subunits. Mutations in Psf1 that disrupt effective engagement result in a more significant role for Polδ (or other polymerases) in synthesizing the leading strand after translesion synthesis (TLS) or repriming (144, 145, 146, 147). In fact, Polε is not even explicitly required for S-phase progression *in vivo* and can be replaced by Polδ, albeit with less efficient synthesis (118). It seems that Polε is flexible and dynamic, responding to the rotational translocation activity of CMG being able to disengage frequently or when encountering lesions. Even so, leading strand damage or replisome blocks that can be overcome by CMG are inhibited or slowed by the presence of Polε (Dpb2) (148, 149). Therefore, Polε is re-recruited, stabilized, and maintained through multicomponent contacts with CMG, Mrc1, and PCNA (150, 151, 152), and those contacts can regulate the ability to traverse genomic blocks and alter the speed of unwinding.

#### CMG–Polα-Primase–Polδ

While Polε extends the leading strand, lagging strand Okazaki fragment synthesis is performed by Polδ (153). Yeast Polδ is a three-subunit complex consisting of catalytic Pol3 and accessory subunits, Pol31 and Pol32. However, Polδ also plays a role in initiating leading-strand synthesis at replication origins in yeast. After priming by Polα-primase, Polδ synthesizes approximately 180 bp of DNA, equivalent to the length of one Okazaki fragment, before Pol ε takes over for continuous replication (154). It is generally thought that Polδ does not physically interact with CMG; however, the Pol32 subunit interacts with the Polα-primase, which is tethered to CMG through the Ctf4 trimer to aid in the stable synthesis of multiple Okazaki fragments (155). Polδ also plays a crucial role in replication termination by taking over leading-strand synthesis from Polε as replication forks converge (156). During this process, Polδ collaborates with the CMG helicase to relieve topological stress caused by supercoiling. Unlike Polε, which is tightly bound to the helicase, Polδ′s independent function allows the DNA between them to rotate more freely, reducing tension and aiding the helicase in unwinding the DNA (156, 157). This coordination is crucial for ensuring the smooth termination of replication, preventing stalling, and completing genome duplication efficiently.

## Causes and consequences of full-on replisome blocks

**Figure 5:**
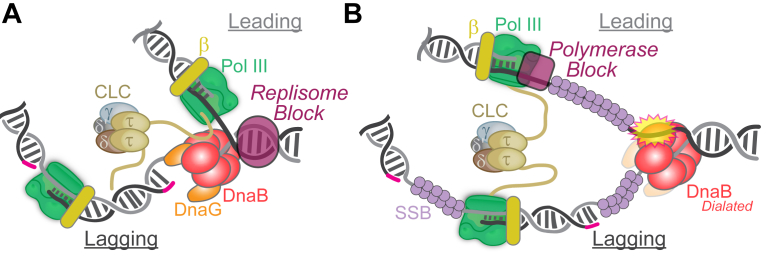
**Genomic blocks leading to replisome stalling or polymerase uncoupling in *E. coli*.** A representation of the *E. coli* replisome responding to (*A*) a major replisome block (*purple oval*) to helicase progression or (*B*) a block only to polymerase progression (*purple rounded square*) resulting in helicase dilation, excluded strand engagement, and uncoupling.

Replication forks may encounter blocks that could lead to whole replisome stalling or fork collapse even under normal conditions from a variety of reasons including RNA transcription conflicts, proteins bound to DNA, or specific template damage (Fig. 5). Any of these could lead to replication fork inactivation requiring restart or recombination to re-establish forks and resume replication in a nonmutagenic manner (158). If blocks are left unresolved, they can lead to gross genomic instabilities, under-replicated regions, and cell death.

### Replication—transcription conflicts

In *E. coli*, the replisome progression rate is 12- to 30-fold greater than that of transcription, thus increasing the chances of replisomes encountering DNA stretches with ongoing transcription (159). If replication and transcription are translocating in the same direction, it is termed codirectional; if they are in the opposite direction, this results in head-on collisions. While head-on collisions will most likely lead to replication fork arrest, codirectional collisions are less severe (160). Therefore, a vast majority of the transcription units, highly transcribed and essential genes, are encoded by the leading strand to favor codirectional collisions that reduce interference of transcription with replication (161).

Based on *in vitro* studies of co-directional collisions, the (RNAP will dissociate from DNA, Pol III will dissociate from the clamp-bound DNA, and the β-clamp will reassemble upstream of RNA-DNA hybrid to re-recruit the Pol III (159, 162). Pol III can then hop onto the newly assembled β-clamp, use the mRNA transcript as the primer-template, and resume synthesis, while DnaB stays bound to the lagging strand. The mRNA primer can be removed later through similar principles of Okazaki fragment maturation. Should the helicase dissociate, PriA, an SF2 superfamily DNA helicase, recognizes abandoned replication forks through a 3′-OH binding pocket of the nascent leading strand at the fork with high affinity, ssDNA/dsDNA of the lagging strand, and parental dsDNA ahead of the fork. In addition to fork substrates, PriA can recognize and bind D-loops or R-loops on the nascent leading strand, and well-positioned 3′-OH termini may also serve as a potential substrate for PriA-dependent replisome restart by reloading DnaB/C followed by Pol III (158, 163, 164, 165, 166).

The T7 replisome operates in a manner similar to that of the *E. coli* replisome, with the concerted action of helicase-polymerase displacing the RNAP and utilizing the RNA transcript as its primer for reinitiation (85). Again, head-on collisions in T7 are more detrimental as the resulting fork arrest and collapse can lead to genome instability. Replication arrest from head-on collisions could result from the built-up torsional strength of positively supercoiled DNA ahead of both transcription and replication or from the physical interaction between the machinery of these processes. However, as most of the stalled fork zones are found near transcribed regions, the latter explanation is more plausible, as supercoiled regions can be mitigated by gyrase (167, 168). Two possible outcomes from replication-transcription conflicts are possible: 1) prolonged stalling can result in fork collapse and eventually double-stranded breaks or 2) forks can be reversed, protected, and restarted though various mechanisms (169, 170). At the onset of these conflicts, RecBCD recombination enzymes (independent of RecA) are essential for the resolution and processing R-loops, while accessory helicases, UvrD or Rep, can remove RNAP and other proteins bound to DNA to help resolve the block (171, 172, 173).

The eukaryotic replisome is also heavily challenged by the presence of transcription and resulting R-loop structures encountered during replication that can trigger a DNA damage response if not resolved (174, 175, 176). Similar to the other systems described above, head-on collisions are also the most deleterious in eukaryotes (177) and require one of many accessory helicases to unwind the R-loop to resume replisome progression (178, 179). Even worse, replication conflicts involve R-loops that are still engaged with the RNAP complex and may require dangerous fork cleavage and religation mechanisms to restart fork progression (180, 181). Alternatively, RNAP can be transiently removed or targeted for degradation by ubiquitination to allow for replisome progression (182, 183). Codirectional transcriptional conflicts on their own are proposed to be easily handled by direct unwinding by CMG, but *in vitro* experiments with dCas9 stabilized R-loops or RNA:DNA G4 structures are able to block both orientations in yeast and require Pif1 to resolve the block (184, 185). It is also possible that CMG bypass of R-loops directly without displacement could allow for a more rapid mechanism of restart by utilizing the RNA transcript as a primer for Polε reengagement (186, 187, 188) as described in the bacterial system (159, 162).

### Strong noncovalent protein-bound conflicts

The strong binding of proteins to DNA has also been shown to act as a barrier to replication by causing complete replisome blockage (Fig. 5*A*). The most effective blocks consist of arrays of binding sites for high-affinity DNA-binding proteins such as the lac-repressor protein, LacI, binding to the *lacO* sequence (189). LacI–*lacO* complexes can halt replication forks from either the 3′ or 5′ direction (190). In *E. coli*, LacI binding creates strong roadblocks to the DnaB helicase, halting its progression and leading to the stalling of both the helicase and the polymerase in a tightly coupled manner (191). The artificial insertion of LacI-*lacO* array sites within chromosome regions has been used to inform on the consequences of artificially halting replisome progression. In eukaryotes, the insertion of LacI-*lacO* arrays efficiently blocks entire eukaryotic replisomes (148, 192). In yeast, the CMG helicase is capable of bypassing protein-mediated barriers, such as LacI-*lacO* or Fob1 at the replication fork barrier (193) and continues unwinding DNA independently of polymerase (148). However, the addition of Polε induces pausing of CMGE at these barriers, suggesting that CMGE coupling ensures proper regulation of helicase activity, maintaining coordinated progression and genome stability during replication. LacR-*lacO* blocks have also been incorporated into the *in vitro Xenopus* egg extract system to better understand replication termination and the converging of forks, showing that CMGs dissociate after the dissolution and ligation of leading and lagging strands (194). Improper replisome decoupling activities can result in the generation of ssDNA or dsDNA breaks and an elevated amount of error-prone DNA repair from various mechanisms (157, 192, 195, 196, 197, 198, 199).

Of course, native termination sites (*Ter*) in the *E. coli* genome bound by the Tus protein create a strong roadblock to stall the DnaB helicase and initiate termination in bacteria (200). Similar to the LacI-*lacO* roadblock, the Tus-*Ter* hub halts fork progression and stalls both the helicase and the polymerase (201). Similarly, bacterial Tus-*Ter* site barriers can be incorporated into mammalian systems (200) and stall replisomes advancing from one side only because of their sequence asymmetry (202). It has been noted that pausing at artificially engineered Tus-*Ter* barriers in yeast is Tof1-checkpoint independent and unaffected by the Rrm3 helicase, perhaps reflecting the high mechanical nature of this barrier (203). The multisequence recognition model is challenged by observing Rrm3-independence of bacterial Tus-*Ter* barriers when transplanted into budding yeast chromosomes (204). However, since Tus-*Ter* can also block helicase-independent unwinding of the DNA helix, they might constitute a unique replication fork barrier type of site (203). Further research in this area will lay the foundation for more detailed studies of the mechanics and consequences of protein barrier pausing on genome integrity.

G4 quadruplex DNA structures can also halt the progression of the CMG helicase by fully blocking and plugging the N-terminal central channel. Interestingly, the resolution of this stalled structure also allowed the researchers to visualize intermediate conformational states that confirmed a helical inchworm-like out-of-plane open (*i.e.,* stepping) DNA translocation model (205) that is very similar to that shown previously for the T7 replisome (82). In order for the replisome to proceed, the G4 must be melted by other accessory helicases. The 3′ end of the G4 is sequestered by CMG, and so, it is likely that DHX36 helicase would be unable to melt this type of trapped G4 (206). Alternatively, 5′ to 3′ helicases, such as Pif1 (207) or FANCJ (208), could aid CMG in tugging on this block from the unblocked side so that the sequence can translocate through the central pore of CMG.

### Covalently crosslinked proteins to DNA

Proteins continuously interact with DNA noncovalently, and while most of these interactions are transient, endogenous metabolism or exogenous factors may covalently trap proteins, forming stable DNA-protein crosslinks (DPCs). These may be caused directly through enzymes acting on DNA, termed enzymatic DPCs (209), or by other proteins crosslinked from aldehydes or radicals, termed nonenzymatic DPCs (210). Both types of DPCs can act as barriers and pose a threat to ongoing DNA replication progression (Fig. 5*A*). If the size of the DPC on the translocating strand is smaller than the central pore of the helicase, then the enzymes can translocate over and through. The size limit of these DPCs is between 5.0 to 14.1 kDa for helicases in the T7, *E. coli*, and eukaryotic systems. With nonbulky translocating strand DPCs, the helicase can traverse the DPC, likely using its ability to dynamically alter its conformation, but the polymerase will still stall, resulting in replisomal uncoupling (Fig. 5*B*). However, if the DPC is on the nontranslocating strand, the respective polymerase on that strand will stall, but the helicase and translocating strand polymerase will maintain coupling. If the DPC on the translocating strand is greater than the threshold for translocation, the helicase and polymerase will stall, resulting in the whole replisome stalling (Fig. 5*A*) and subsequently dissociate, initiating fork arrest (211).

**Figure 6:**
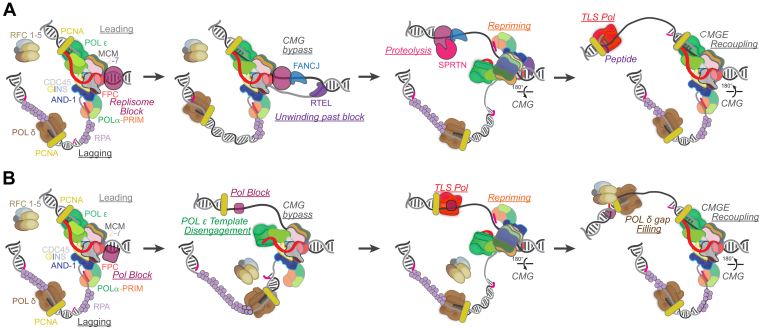
**Genomic blocks can be traversed by CMGE to reinitiate synthesis downstream in eukaryotes.***A*, eukaryotic metazoan replisome (colored as in Fig. 1*C*) encountering a full replisome block can utilize alternative helicases, RTEL and FANCJ, to unwind the parental duplex downstream of the block. FANCJ then recruits SPRTN protease to degrade the protein block. CMG can open its ring structure, bypass the block by dragging along a disengaged POLε through interactions with the fork protection complex (FPC) as it alters its conformation to allow the traversing of the block, and reinitiate priming downstream by POLα-PRIM. Finally, POLε reengages the leading strand and the remaining peptide can be bypasses by TLS polymerases. *B*, alternatively, a lesion can be a block only to POLε. Similarly, POLε can disengage from the template and remain bound to CMG to traverse of the block, reprime downstream, bypass damage by TLS polymerases and POLδ gap filling, while POLε reengages the leading strand. CMG, CDC45, MCM2-7, GINS; CMGE, CMG polymerase epsilon; TLS, translesion synthesis.

Multiple repair pathways exist to resolve these DPCs. In both bacteria and eukaryotes, both nucleotide excision repair (NER) and homologous recombination (HR) pathways play a role in repairing the DPCs. In bacteria, if the DPC is less than 12 to 14 kDa, NER is the dominant pathway, but for protein crosslinks greater than this size, RecBCD-mediated HR pathway predominates (212). In contrast to bacteria, mammalian NER is not responsible for repairing DPCs. Instead, HR was thought to be the predominant pathway in tolerating DPCs (213). In yeast and *Xenopus*, DPCs can be proteolyzed into a smaller peptide that can be translocated by the helicase but stall the polymerase (*decoupling*), and then synthesized over by a specialized TLS polymerase before reassembling Polε with CMG (*recoupling*) (198, 214). Should the entire metazoan CMG replisome become stalled, DPC bypass can be facilitated by additional helicases, including RTEL or FANCJ, which are able to unwind the parental duplex in front of the block using 3′ to 5′ and 5′ to 3′ unwinding activities, respectively (215, 216, 217) (Fig. 6*A*). Once the upstream parental duplex is unwound by RTEL, CMG is thought to crack open one of the MCM interfaces to allow for the passage of the DPC and reengagement around the leading strand. FANCJ working in the opposite direction may also facilitate unwinding, but it is required to recruit the SPRTN protease to begin degrading the DPC (218). Afterward, the remaining crosslinked small peptide can be bypassed by TLS polymerases, POL η or POL κ (219).

## Lesions that decouple synthesis from unwinding

### Polymerase stalling lesions in the bacteriophage T7 system

In T7 phage, helicase translocation is unaffected by lesions on the leading strand as it encircles and moves along the lagging strand. While some bulky adducts on the lagging strand may impede gp4 helicase movement, it can translocate over those that fit through the central channel of the helicase, including cyclobutane pyrimidine dimers (220, 221). The leading strand gp5 polymerase is incapable of moving past the lesion on its own, but when coupled with the gp4 helicase, they pause together, and the increased dwell time helps the complex overcome the lesion and move forward (221). Therefore, when gp5 has difficulty incorporating nucleotides opposite a lesion, it is likely that gp4 keeps the polymerase tethered to the substrate, increasing the residence time, until successful incorporation can happen. When the helicase and polymerase speeds were selectively altered to examine impacts on coupling, the polymerase did not outpace the helicase when it was slowed, but rather, they stayed functionally coupled. However, if the leading strand polymerase stalls significantly, the helicase does continue to unwind, leading to functional uncoupling in more severe cases (84). Therefore, in T7, the concerted action of helicase and polymerase is beneficial in overcoming lesions that could otherwise cause fork stalling or collapse, as T7 does not possess specialized TLS polymerases that could otherwise fulfill this role.

### Polymerase stalling lesions in the *E. coli* system

In bacteria, when the replisome encounters lesions on the leading strand, helicase progression is unaffected as it translocates along the lagging strand. However, the leading strand polymerase does stall at lesions, including bulky adducts, abasic sites, or cyclobutane pyrimidine dimers (222). When polymerase stalling occurs, there are three main pathways to resolve these blocks: 1) lesion skipping and repriming, 2) TLS substitutions, or 3) template switching (223, 224).

When Pol III encounters lesions on the leading strand, it is unable to synthesize past this lesion, causing it to stall. This Pol III stalling will decouple the helicase and polymerase. Interestingly, when decoupled, the unwinding speed of DnaB is reduced by ∼80%, likely through hexamer dilation and engagement of the excluded strand, thus allowing Pol III to recouple once it bypasses a lesion, effectively reducing the build-up of long stretches of intervening ssDNA. This passive fail-safe mechanism adopted by *E. coli* has been referred to as a “*dead-man’s switch*” to minimize uncoupling and limit SOS induction (225). Dissociation of τ appears to be playing a role in regulating helicase speed by inducing a conformation change in DnaB toward a dilated state, which can then engage the excluded strand and slow unwinding (Fig. 5*B*) (4).

In another model for Pol III stalling, the helicase would remain tethered to the polymerase through τ, facilitated by the long unstructured linker of domains IV and V, but any upstream unwinding by DnaB would form a loop of ssDNA (222). The replisome would remain bound to the template but reinitiate synthesis downstream of the lesion, facilitated by DnaG-dependent repriming. If repriming downstream of the lesion is efficient and close to the damaged site, the stalled polymerase can be recycled onto the new primer-template junction; however, lesion skipping in this manner would lead to the generation of ssDNA gaps of various sizes, later repaired by Pol I–dependent gap filling, RecA-dependent HR employing the RecFOR pathway, or template switching (224). While the replisome has an inherent system to tolerate some lesions in a nonmutagenic way, more problematic lesions leading to more severe decoupling may produce long stretches of ssDNA bound by RecA that induce the mutagenic SOS response (3, 226).

*E. coli* possesses three TLS polymerases: Pol II, IV, and V. Of these, Pol II and IV are expressed in cells at low concentrations under normal physiological conditions and are only upregulated when induced by the SOS response (227, 228). Pol V expression occurs during later stages of the SOS response (229). Pol II and IV can switch with Pol III at stalled sites where the β-clamp is bound to both polymerases. Although the TLS Pols II and IV are not known to interact with DnaB, these alternative replisomes are stable but progress at slower speeds. The helicase adapts its conformation to a dilated state, slowing unwinding and maintaining a connection with slower synthesis to prevent the buildup of ssDNA gaps (2). Therefore, during greater levels of DNA damage, slower progressing replisomes provide time for DNA damage tolerance, which gives a greater probability of replisome recoupling after TLS (230, 231).

Lesions occurring on the lagging strand are less problematic for the *E. coli* replisome, primarily from the distributive nature of the lagging-strand synthesis. Studies conducted with abasic lagging-strand lesions have shown that leading-strand synthesis is unaffected and that subsequent lagging-strand synthesis continues only with the lesion to be repaired later (232). In instances where the lagging-strand polymerase is blocked, the leading strand polymerase will continue to be coupled with the helicase while still being attached to the lagging-strand polymerase, but the two enzymes will be functionally uncoupled. SSB-coated ssDNA was less likely to cause the polymerase to dissociate from the β-clamp and DNA, but upon SSB exhaustion, the free ssDNA helped release the polymerase from the blocked site to synthesize new Okazaki fragments to ensure replication fork progression despite the presence of lagging-strand lesions (232).

### Template damage and CMGE plasticity in the eukaryotic system

When the eukaryotic replisome encounters a roadblock that does not distort the double helix, such as intrastrand adducts or abasic sites, the coupling response depends on whether the lesion is on the leading or lagging strand. If the lesion is present in the lagging strand, it may stall POL α-primase or POL δ but does not affect replisome progression or DNA synthesis because of rapid repriming on the lagging strand by a new Pol α-primase, which eventually facilitates bypassing the lesion. The gaps formed are later repaired or filled in by TLS polymerases postreplicatively (233). If the lesion is present in the leading strand, POL ε halts progression typically without hindering helicase activity (234) (Fig. 6*B*). The MCM ring's central channel can accommodate duplex DNA, allowing the helicase to continue moving even when damaged nucleobases, abasic sites, or bulky adducts are present in the leading-strand template, without causing a steric block to unwinding (149, 217, 233, 235, 236, 237). This disruption can lead to polymerase-helicase uncoupling and slow the progression of the replication fork. To overcome leading-strand lesions and restart fork progression, the replisome employs three main pathways: PRIMPOL-dependent repriming, TLS, and fork reversal to bypass the lesion and maintain the continuity of DNA replication (234, 238).

While decoupling in bacteria shows a complete physical disengagement from helicase and polymerase, mechanistic studies in yeast propose that eukaryotic Polε remains physically tethered to the CMG helicase during decoupling, through interactions involving Psf1 as a hinge (Fig. 6*B*) (116). Similarly, in humans, POLE2 N-terminal helical domain and PSF1 enable POLε to dissociate from MCM subunits while remaining tethered to CMG (124). When POLε uncouples from leading-strand synthesis upon encountering DNA lesions, POLδ can take over and synthesize past the lesion, filling in any gaps. This ensures the continuity of replication despite the presence of the lesion. TLS polymerases can also be recruited to bypass the damage, allowing the replication fork to move forward and maintain genomic integrity. Tethering of POLε to CMG facilitates rapid re-engagement of POLε when the lesion is cleared, ensuring minimal disruption to the replication process (116). The dynamic decoupling and recoupling of leading-strand synthesis and DNA unwinding in eukaryotes is crucial for effective replication under challenging conditions, thereby maintaining genome integrity in spite of purported blocks. It is more appropriate to describe this process as a dynamic interaction, rather than a strict decoupling, as observed in bacteria.

Although CMG helicase DNA unwinding activity is not halted during Polε disengagement, cells adopt mechanisms to control excessive DNA unwinding by CMG at the replication fork and prevent fork collapse. Replisome studies in yeast suggest that the Rad53 kinase plays a crucial role in regulating excessive CMG unwinding. Although the precise molecular mechanism remains unclear, Rad53 likely restricts CMG activity by targeting Mcm10 to prevent an excess of ssDNA production and replication protein A exhaustion, allowing for unprotected nuclease degradation (239, 240). Interestingly in budding yeast, the Cdc45 subunit of the CMG helicase has been shown to have ssDNA binding affinity (241). When Polε is halted at a lesion, CMG will continue unwinding DNA, but when a stretch of >40 nt of ssDNA is formed, Cdc45 binds the excluded strand and halts the helicase progression, similar to the steric exclusion and wrapping model proposed for bacterial and archaeal helicases (103, 242, 243). In vertebrate systems (*Xenopus*), functional uncoupling of helicase and polymerase activates the ATR checkpoint kinase from excess accumulation of ssDNA that is amplified further upon ATR-interacting protein binding (244). However, excess unwinding alone is not sufficient for ATR checkpoint activation; DNA synthesis by POLα is also necessary to create the primer-template junction, which is essential for recruiting the RAD9–HUS1–RAD1 complex to fully activate the checkpoint (244). This cascade leads to the phosphorylation of CHK1 and other downstream effectors, initiating cell cycle arrest and repair mechanisms to ensure that helicase activity does not proceed unchecked, thus preventing replication fork collapse.

Given the role of the CMG helicase in coupling Polε to the leading-strand synthesis and progressing replication, genetic mutations within the components of CMG can lead to its decoupling from the replication machinery, resulting in cellular defects, cancer, immunity, and neurological diseases (245, 246, 247). CMG-POLε components are therefore considered caretakers of the genome. When GINS subunit PSF1 is mutated, DNA replication becomes impaired, triggering replication stress, defective checkpoint signaling, and genomic instability (248). For example, heterozygous mutations in GINS1 revealed intrauterine growth retardation, chronic neutropenia, and NK cell deficiency, all associated with defective DNA replication (249). Another study showed human dermal fibroblasts lacking PSF1/PSF2 resulted in delays in DNA replication, impaired chromatin binding of CDC45 and PCNA, and accumulation of DNA damage markers like γH2AX and phosphorylated CHK2 (250). These defects may be caused by the loss of proper coupling, leading to replication stress and genomic instability, emphasizing the critical role of GINS in maintaining replication fidelity and chromosomal integrity. A study conducted in yeast showed that a mutation of the *psf1-1* allele disrupted CMG-mediated targeting of POLε to the leading strand, causing POLδ to take part in leading-strand synthesis, both *in vitro* and *in vivo* (144). This indicates that the proper functioning of the CMG–GINS complex is crucial for ensuring the division of labor between POLε and POLδ on the leading and lagging strands. It also highlights the importance of the replisome coupling process in maintaining accurate DNA replication and avoiding consequences of replication-associated pathologies resulting from decoupling.

## Conclusion

The dynamic coupling and decoupling of the helicase and polymerase enzymes is a necessary process to ensure high-fidelity DNA replication and genomic stability in response to innumerable challenges. While the core replication machinery plays a significant role in the main enzymatic functions in the replisome, associated accessory proteins dynamically regulate and stabilize the core complexes to govern these processes. Despite the evolutionary differences between phage, bacteria, and eukaryotes, all replication systems converge on the fundamental principle of ensuring efficient coupling while employing mechanisms to minimize decoupling in response to DNA damage or replication stress. While simple systems, such as T7 and bacteria, have low to moderate coupling and decoupling complexities with repetitive dynamic associations and structural alterations, eukaryotes have more complex mechanisms with several intermediates and multiprotein connections that ensure tight tethering to reduce any checkpoint activation. This review highlights the similarities and differences that exist between these three model systems. Understanding how these core and associated proteins work in concert presents a broader picture of not only coupling and decoupling mechanisms but also the intricate regulation and adaptation of DNA replication across different levels of organisms. Future research involving structural biology of intermediate complexes, advanced biochemical reconstitution techniques, and unique cellular screens will provide a more comprehensive understanding of the plasticity of coupling and decoupling mechanisms. This will be crucial for understanding replication-associated diseases and thereby pave the way to develop novel therapeutic strategies that target decoupling as an inhibition strategy. Already, current chemotherapeutic agents cause genomic instabilities that challenge or decouple the replisome, activate cellular checkpoints, and cause double strand beaks. Future therapeutic strategies could take a more moderate approach by targeting the conformational states and interactions within the replisome. Such approaches may involve modulating ubiquitin ligases that mark stalled, decoupled, or terminating replisomes (251, 252, 253) or deubiquitinating enzymes involved in maintaining normal replisome progression (254).

## Conflict of interest

The authors declare that they have no conflicts of interest with the contents of this article.
